# Comprehensive Characterization of Androgen-Responsive circRNAs in Prostate Cancer

**DOI:** 10.3390/life11101096

**Published:** 2021-10-15

**Authors:** Zhe Kong, Yali Lu, Xuechao Wan, Jun Luo, Dujian Li, Yan Huang, Chenji Wang, Yao Li, Yaoting Xu

**Affiliations:** 1 Shanghai Engineering Research Center of Industrial Microorganisms, School of Life Science, Fudan University, Shanghai 200433, China; 18110700042@fudan.edu.cn (Z.K.); 17210700062@fudan.edu.cn (Y.L.); 15210700108@fudan.edu.cn (X.W.); huangyan@fudan.edu.cn (Y.H.); chenjiwang@fudan.edu.cn (C.W.); 2 Department of Urology, Shanghai Fourth People’s Hospital Affiliated to Tongji University School of Medicine, Shanghai 200434, China; abell_luojun@sina.com (J.L.); djl00777@163.com (D.L.)

**Keywords:** prostate cancer, androgen-responsive circRNA, WTAP, TNRC6, *circNFIA* and *circZNF561*

## Abstract

The androgen receptor (AR) signaling pathway plays an important role in the initiation and progression of prostate cancer. Circular RNAs (circRNAs), the novel noncoding RNAs without 5′ to 3′ polarity or 3′ poly (A), play an important role in multiple diseases. However, the potential roles of androgen-responsive circRNAs in prostate cancer remain unclear. In this study, we identified 3237 androgen-responsive circRNAs and 1954 androgen-responsive mRNAs after dihydrotestosterone (DHT) stimulation using microarray. Among them, the expression of 1296 androgen-responsive circRNAs was consistent with that of their parent genes, and we thought AR might regulate the expression of these circRNAs at the transcriptional level. In addition, 1941 circRNAs expression was not consistent with their parent genes, and we speculated that AR may regulate the expression of those circRNAs at the posttranscriptional level through affecting alternative splicing. Analyzing the androgen-responsive circRNAs regulated at the posttranscriptional level, we identified two key RNA binding proteins (RBPs), WTAP and TNRC6, using the circInteractome database, which may play important role in the biogenesis of androgen-responsive circRNAs. Furthermore, we explored the potential biological functions and predicted the molecular mechanisms of two dysregulated circRNAs (*circNFIA* and *circZNF561*) in prostate cancer. In this study, we revealed that *circNFIA* was upregulated in prostate cancer tissues and plasma samples from patients with prostate cancer; *circNFIA* may play an oncogenic role in prostate cancer. In contrast, *circZNF561* was downregulated and may act as a tumor suppressor in prostate cancer. Our results suggest that androgen-responsive circRNAs might regulate the progression of prostate cancer and could be novel diagnostic biomarkers.

## 1. Introduction

Prostate cancer is the second most frequently diagnosed cancer in men worldwide, and 1.4 million estimated cases were diagnosed in 2020 [1]. The incidence rates of prostate cancer increased in most countries, such as Sweden, Thailand, China, and Lithuania [2]. Prostate cancer is hormone-responsive, and castration leads to tumor regression in prostate cancer patients [3]. Androgen deprivation therapy (ADT) is the main treatment for advanced and metastatic prostate cancer [4]. However, almost all patients develop castration-resistant prostate cancer (CRPC) approximately 2–3 years after ADT, at which time, the serum PSA levels increase again and the disease rapidly worsens [5,6].

The androgen receptor (AR) signaling pathway plays a vital role in the diagnosis and treatment of prostate cancer. CRPC, previously defined as hormone-independent prostate cancer, is now known to still be androgen dependent. AR amplification [7], AR mutations [8], mutations in coactivators or corepressors [9,10,11], androgen-independent AR activation [12], and alternative androgen production [13,14] contribute to the progression of CRPC. Understanding the role of the AR signaling pathway in the progression of prostate cancer is important to develop future therapies.

With advances in high-throughput sequencing technologies and bioinformatics, a large number of circRNAs have been gradually discovered in mammalian cells [15]. CircRNAs, a novel type of noncoding RNAs with a covalent closed-loop structure, are derived from pre-mRNAs via back-splicing and have no 5′ cap or 3′ poly (A) [16,17]. CircRNAs are more stable and protected against RNA exonucleases in vivo. Accordingly, recent studies have suggested that circRNAs could be promising biomarkers in multiple diseases [18]. In addition, an increasing number of studies have found that circRNAs play important roles in diseases, such as diabetes [19], cardiovascular disease [20], chronic inflammatory diseases [21], and neurological diseases [22], especially in a variety of tumors. For example, *circSMARCA5* acts as a tumor suppressor in liver cancer [23], malignant glioma [24], non-small cell lung cancer [25], and plays an oncogenic role in prostate cancer [26]. CircRNAs act as microRNA sponges to regulate the development and progression of disease. For example, *CDR1as*, also called *ciRS-7*, was reported to have approximately 70 conserved *miR-7* binding sites and to act as a potential *miR-7* sponge to contribute to several biological processes [27,28]. Recently, our group also found that *circFOXO3* could promote prostate cancer progression by competitive binding of *miR-29a-3p* [29]. However, the biogenesis and biological functions of androgen-responsive circRNAs in prostate cancer still need to be further explored.

In this study, we identified circRNAs as diagnostic and therapeutic markers and explored the potential functions of circRNAs in prostate cancer. We identified a set of androgen-responsive circRNAs and two important regulatory factors, WTAP and TNRC6, which may involve in circRNA biogenesis. In addition, we explored the potential biological functions and predicted the molecular mechanism of two androgen-responsive circRNAs (*circNFIA* and *circZNF561*), which may act as promising biomarkers for the diagnosis and treatment of prostate cancer.

## 2. Materials and Methods

### 2.1. Human Prostate Cancer Samples

Prostate cancer patient tissue samples and matched adjacent normal samples were obtained from patients who underwent radical prostatectomy at Fudan University Shanghai Cancer Center. Serum samples from newly diagnosed prostate cancer patients (*n* = 26) and healthy donors (*n* = 19) who provided informed consent were obtained at Center named above. These patients did not receive any preoperative treatment. The study was approved by the Research Ethics Committee of Fudan University Shanghai Cancer Center. All the samples were collected and used for gene expression analysis by qPCR.

### 2.2. Microarray Analysis

LNCaP cells were starved for 3 days and treated with 100 nM DHT for 8 h. Then, the cells were collected, and microarray analysis was performed as previously described [30] (Shanghai Biotechnology Corporation, Shanghai, China). In brief, total RNA was amplified, labeled with a Low Input Quick Amp Labeling Kit and then purified using RNeasy mini kit. Each slide was hybridized with 1.65 μg Cy3-labeled RNA for 17 h and then scanned by an Agilent microarray scanner (Agilent technologies, Santa Clara, CA, USA). The data were extracted using Feature Extraction software 10.7 (Agilent Technologies). The raw data were normalized by the quantile algorithm and limma package in R (Version 3.6.2).

### 2.3. Cell Culture and Treatment

LNCaP, DU145, 22Rv1, PC-3, and WPMY-1 cells were purchased from the American Type Culture Collection (ATCC, Manassas, VA, USA). All the cells were cultured in RPMI-1640 or DMEM medium supplemented with 10% fetal bovine serum (ExCell Bio, Shanghai, China). The cells were maintained in an incubator at 37 °C containing 5% CO_2_. The androgen treatment assays were performed as described previously [31]. Briefly, LNCaP cells were starved for 3 days and then treated with DHT. The cells were collected and used in the following detection.

### 2.4. RNA extraction and qPCR

Total RNA was isolated from homogenized prostate cancer tissue or cultured cells using MagZol Reagent (Magen, Guangzhou, China) as previously described [29]. Total RNA was reverse transcribed using the PrimeScript^™^ RT reagent Kit (Takara, Japan) according to the manufacturer’s protocol. The relative expression of candidate genes was determined using AceQ qPCR SYBR Green Master Mix (Vazyme, Shanghai, China) on LightCycler^®^ 480II (Roche, Basel, Switzerland). The primers used for the qPCR analysis are listed in Supplementary Table S1.

### 2.5. Transfection

Cells were seeded in 6-well plates until cell confluence reached 60%–80%. Liposomal cocktails with siRNA (GenePharma, Shanghai, China) (50 nM final) were generated with HilyMax (Dojindo Laboratories, Kumamoto, Japan) in Opti-MEM (Invitrogen, Carlsbad, CA, USA) according to the protocol. The transfected cells were incubated for 48 h and then used for gene expression analysis or other experiments. The sequences of the specific siRNAs are shown in Supplementary Table S1.

### 2.6. Cell Apoptosis

Cells were cultured in 6-well plates after transfection. Cell apoptosis was measured using the Annexin V-FITC apoptosis kit (Dojindo Laboratories, Kumamoto, Japan). After incubation at room temperature for 15 min, cell apoptosis was analyzed on a FACSCalibur flow cytometer (Becton, Dickinson and Company, Franklin Lakes, NJ, USA).

### 2.7. Cell Proliferation

Cell Counting Kit-8 (Dojindo Laboratories, Kumamoto, Japan) was used to assess cell proliferation according to the manufacturer’s protocol. Briefly, transfected cells were seeded in 96-well plates and cultured in 100 μL medium. 10 μL of CCK-8 solution was added to each well at 0, 24, 48, and 72 h and further incubated for 2 h to allow the colorimetric reaction to occur. The absorbance at 450 nm was measured by a Microplate Reader ELx808 (Bio-Tek, Winooski, VT, USA) to determine cell viability.

### 2.8. Cell Cycle

Transfected cells were cultured in 6-well plates for 48 h. Then, the cells were collected and treated with Triton X-100 (0.03%, Sigma, Darmstadt, Germany) and propidium iodide (PI, 50 ng/mL, Beyotime, Shanghai, China) for 15 min. Cell cycle analysis was performed by a FACSCalibur flow cytometer (Becton, Dickinson and Company, Franklin Lakes, NJ, USA).

### 2.9. Construction of ceRNA Network

The circBank (http://www.circbank.cn/ (accessed on 18 September 2021)) was used to predict miRNA binding sites. We found 59 miRNAs targeted to *circNFIA* and 26 miRNAs targeted to *circZNF561*. The networks were drawn using Cytoscape 3.0. Following this, we identified the target genes of miRNAs interacting with *circNFIA* or *circZNF561* using the miRDB database. The target score ≥ 90 was chosen to construct circRNA-miRNA-mRNA networks using Cytoscape 3.0.

### 2.10. Statistical Analysis

The numerical data are presented as the mean ± standard deviation (SD) of at least three determinations. Statistical comparisons between groups of normalized data were performed using *t*-tests or Mann–Whitney *U*-tests according to the test conditions. A *p* < 0.05 was considered statistically significant with a 95% confidence level.

## 3. Results

### 3.1. Identification of Androgen-Responsive circRNAs in Prostate Cancer

**Figure 1:**
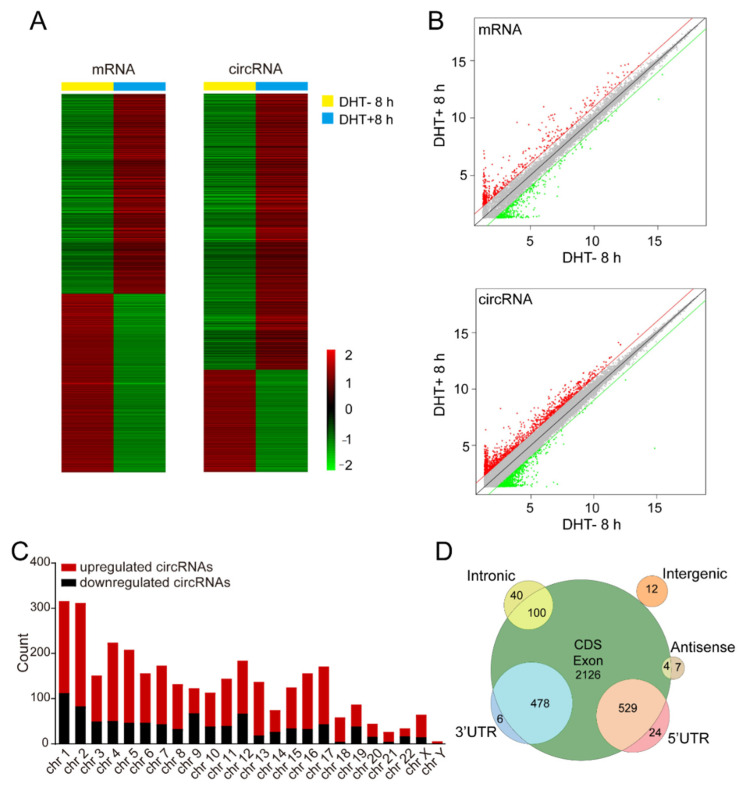
Identification of androgen-responsive circRNAs in prostate cancer. (**A**) The heatmap for androgen-responsive mRNAs and circRNAs in LNCaP cells after 8 h of 100 nM DHT treatment. (**B**) The volcano plot for androgen-responsive mRNAs and circRNAs. (**C**) The statistical analysis of androgen-responsive circRNAs arranged by chromosome. (**D**) The classification of androgen-responsive circRNAs according to their genomic localization.

To identify the differentially expressed mRNAs and circRNAs after DHT stimulation, we performed high-throughput microarray analysis and the expression of 88,751 circRNAs and 18,854 mRNAs was detected. Of these, 3237 circRNAs and 1954 mRNAs were differentially expressed. As shown in Figure 1A,B, 2295 circRNAs and 969 mRNAs were upregulated and 942 circRNAs and 985 mRNAs were downregulated after DHT stimulation. Interestingly, the number of upregulated circRNAs was approximately 2.5 times that of downregulated circRNAs, while the number of upregulated mRNAs was close to that of downregulated mRNAs. The result suggests that the AR signaling pathway may have differences regulating the expression of mRNAs and circRNAs and circRNAs may not be meaningless splicing byproducts.

We also analyzed the distribution of the circRNAs on the human chromosomes and we found that the number of upregulated circRNAs derived from each chromosome was greater than the number of downregulated circRNAs (Figure 1C). In addition, most circRNAs were derived from exons (CDS region, 3′UTR, and 5′UTR) and a few circRNAs were derived from introns, intergenic regions, and antisense strands (Figure 1D).

### 3.2. Verification of Androgen-Responsive circRNAs

**Figure 2:**
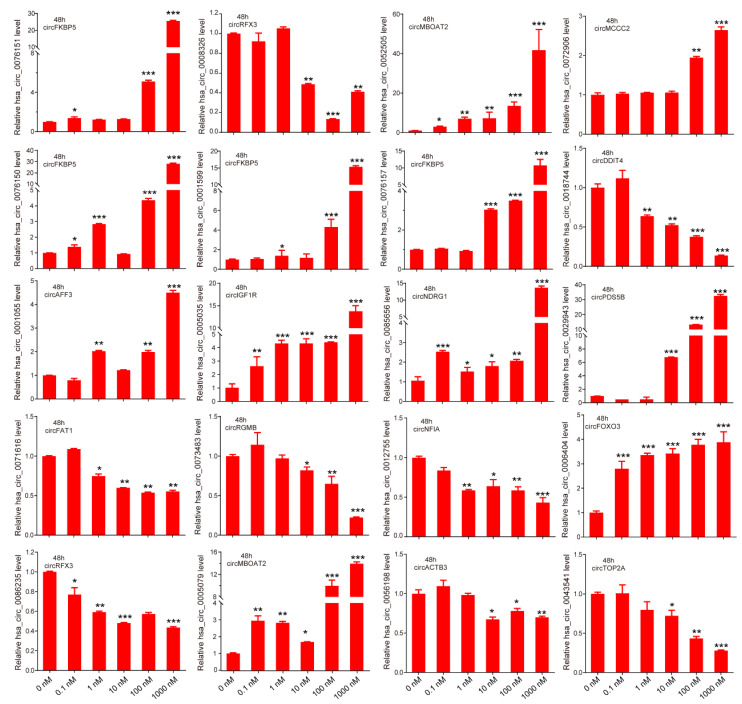
Verification of androgen-responsive circRNAs. The expression levels of 20 circRNAs in LNCaP after different doses of DHT treatment for 48 h were detected by qPCR. Data are mean ± SD. The *p*-value was calculated by the unpaired two-tailed Student’s *t*-test. * *p* < 0.05, ** *p* < 0.01, *** *p* < 0.001.

To verify the expression of androgen-responsive circRNAs, we quantified part of the androgen-responsive circRNAs expression in LNCaP cells after treatment with different doses of DHT (0, 0.1, 1, 10, 100, and 1000 nM) by qPCR. Among them, 12 of 20 circRNAs (*hsa-circ-0076151*, *hsa-circ-0076150*, *hsa-circ-0001055*, *hsa-circ-0001599*, *hsa-circ-0005035*, *hsa-circ-0005079*, *hsa-circ-0052505*, *hsa-circ-0076157*, *hsa-circ-0085656*, *hsa-circ-0072906*, *hsa-circ-0029943*, and *hsa-circ-0006404*) were significantly upregulated and 8 of 20 circRNAs (*hsa-circ-0086235*, *hsa-circ-0008326*, *hsa-circ-0056198*, *hsa-circ-0018744*, *hsa-circ-0043541*, *hsa-circ-0071616*, *hsa-circ-0073483*, and *hsa-circ-0012755*) were significantly downregulated after DHT stimulation (Figure 2). The expression of those circRNAs were consistent with the microarray data.

### 3.3. Characteristics of Androgen-Responsive circRNA Expression

**Figure 3:**
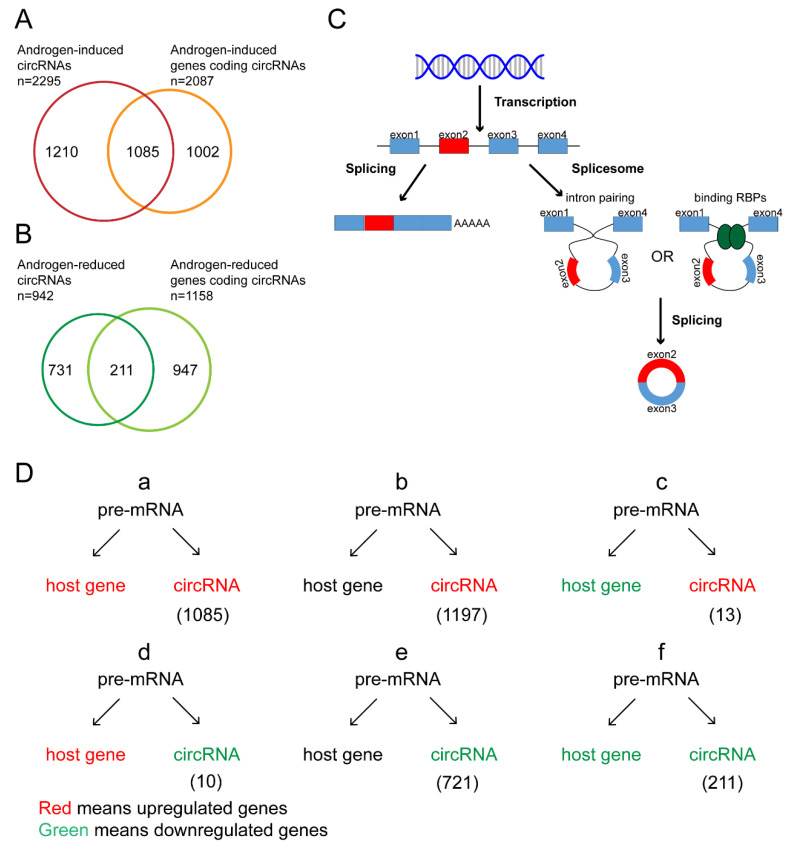
Classification of androgen-responsive circRNA expression. (**A**,**B**) The Venn diagrams for androgen-induced genes encoding androgen-induced circRNAs and androgen-reduced genes encoding androgen-reduced circRNAs. (**C**) The schematic diagram for the biogenesis of mRNA and circRNA. (**D**) The classification of androgen-responsive circRNAs according to the expression of circRNAs and their parent genes.

Both mRNAs and circRNAs are derived from pre-mRNA by alternative splicing. We analyzed the expression of circRNAs and their parent genes in the microarray data. The results showed that only 51.99% of the 2087 circRNAs derived from androgen-upregulated mRNAs were upregulated and 18.22% of 1158 circRNAs derived from androgen-downregulated mRNAs were downregulated (Figure 3A,B). The results showed that only part of the circRNAs derived from androgen-induced or -reduced genes were upregulated or downregulated after DHT treatment, which suggested that the circRNAs and their parent genes had different regulated patterns.

According to the expression of mRNAs and circRNAs after DHT treatment, we divided androgen-responsive circRNAs into six groups (Figure 3D). We focused on the circRNAs in groups a and d that were regulated by androgen at the transcriptional level and the circRNAs in groups b and e that were regulated by androgen at the posttranscriptional level. Among them, the expression of *hsa-circ-0076151*, *hsa-circ-0001055*, *hsa-circ-0005035*, *hsa-circ-0005079*, and *hsa-circ-0073627* was increased after DHT treatment (group a) (Figure 2), while *hsa-circ-0008326* expression was decreased after DHT treatment (group d) (Figure 2).

### 3.4. Androgen Regulated circRNAs at tRanscriptional Level

**Figure 4:**
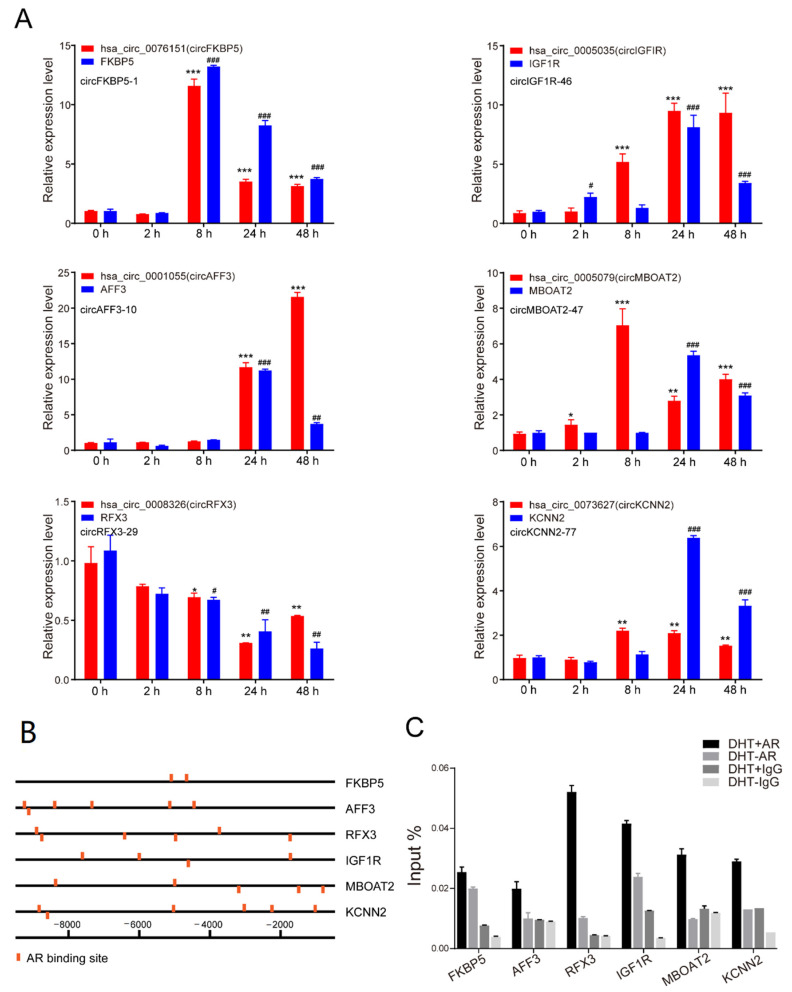
Androgen-regulated circRNAs at the transcriptional level. (**A**) qPCR analysis of the expression of *hsa-circ-0076151*, *hsa-circ-0001055*, *hsa-circ-0005035*, *hsa-circ-0005079*, *hsa-circ-0008326*, *hsa-circ-0073627*, and their parent genes at a series of time points after 10 nM DHT treatment. Data are mean ± SD, The *p*-value was calculated by the unpaired two-tailed Student’s *t*-test. * *p* < 0.05, ** *p* < 0.01, *** *p* < 0.001. # *p* < 0.05, ## *p* < 0.01, ### *p* < 0.001 (mRNAs). (**B**) Prediction of AREs within 10 kb upstream of the transcription start site of *FKBP5*, *AFF3*, *RFX3*, *IGF1R*, *MBOAT2*, and *KCNN2* using hTFtarget. (**C**) ChIP-qPCR showed that AR peaks in these circRNA loci significantly increased after DHT treatment.

To further verify the expression levels of circRNAs and their parent genes, we detected the expression of the above six circRNAs (in groups a and d) and their parent genes at a series of time points after DHT treatment (Figure 4A). The results showed that the expression level of these six circRNAs was basically the same as that of their parent genes. The expression of *hsa-circ-0076151*, *hsa-circ-0001055*, *hsa-circ-0005035*, *hsa-circ-0005079*, *hsa-circ-0073627*, and their parent genes *FKBP5*, *AFF3*, *IGF1R*, *MBOAT2*, *KCNN2* was increased after DHT treatment, while *hsa-circ-0008326* and *RFX3* expression was decreased.

To test the hypothesis that the expression of these six circRNAs were transcriptionally regulated by androgen, we predicted the AREs within the upstream 10 kb from the transcription start site using the hTFtarget database. As shown in Figure 4B, we found many AR binding sites in those genes’ promoters. Furthermore, a ChIP-qPCR assay was performed using anti-AR antibodies to pull down DNA fragments with AREs in LNCaP cells with or without DHT stimulation. Consistently, AR was directly associated with the *FKBP5*, *AFF3*, *RFX3*, *IGF1R*, *MBOAT2*, and *KCNN2* promoters after DHT treatment (Figure 4C). The results showed that AR might directly regulate the expression of *FKBP5*, *AFF3*, *RFX3*, *IGF1R*, *MBOAT2*, *KCNN2*, and their corresponding circRNAs.

### 3.5. Androgen Posttranscriptional Regulated circRNA Expression

**Figure 5:**
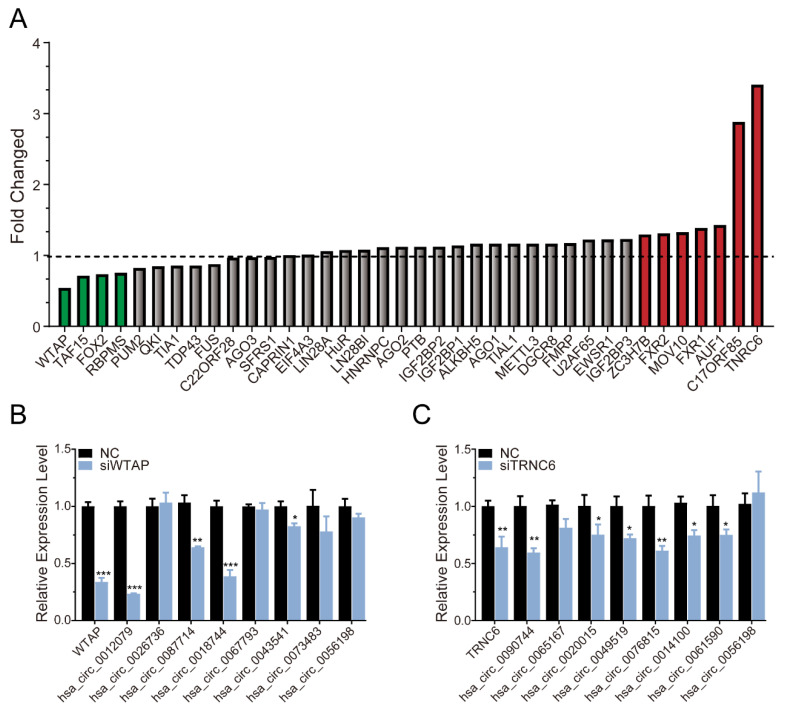
Identification of RBPs involved in regulating circRNA biogenesis. (**A**) Calculation of the ratio of each RBP interacting with androgen-induced circRNAs and androgen-reduced circRNAs. (**B**) Analysis of the expression of circRNAs potentially interacting with WTAP after silencing WTAP. (**C**) Analysis of the expression of circRNAs potentially interacting with TNRC6 after siTNRC6. The *p*-value was calculated by the unpaired two-tailed Student’s *t*-test. * *p* < 0.05; ** *p* < 0.01; *** *p* < 0.001 (circRNAs).

We identified a large number of differentially expressed circRNAs, but their host genes were not differentially expressed (group b and group e). We speculated that the androgen may affect the alternative splicing and regulate these circRNAs expression in the posttranscriptional level. To further explore the regulation of circRNA expression, we firstly identified the RBPs that interacted with the circRNAs in group b and group e using CircInteractome. Then, we calculated the coefficient μ, where

(μ=number of androgen-induced circRNA interacted with a specific RBP(aRBP)Total number of androgen-induced circRNA(a)number of androgen-reduced circRNA interacted with a specific RBP(bRBP)Total number of androgen-induced circRNA(b)) 

of each RBP. The higher the μ value was, the more likely it was that the RBPs interacted with androgen-induced circRNAs. Conversely, the smaller the μ value was, the more likely it was that the RBPs interacted with androgen-reduced circRNAs (Figure 5A). The results showed that WTAP, TAF15, FOX2, and RBPMS potentially regulated the androgen-downregulated circRNAs, while TNRC6, C17ORF85, AUF1, FXR1, MOV10, FXR2, and ZC3H7B potentially regulated the androgen-upregulated circRNAs. Among those RBPs, WTAP, and TNRC6 were the most significant (Figure 5A). Next, we detected the expression of circRNAs potentially associated with WTAP or TNRC6 after knockdown of WTAP or TNRC6, respectively. As shown in Figure 5B, after knockdown of WTAP, the expression of *hsa_circ_0012079*, *hsa_circ_0087714*, and *hsa_circ_0018744* was significantly decreased. Similarly, after knockdown of TNRC6, the expression of *hsa_circ_0090744*, *hsa_circ_0076815*, *hsa_circ_0049519*, *hsa_circ_0014100*, and *hsa_circ_0061590* was significantly decreased (Figure 5C). We initially revealed that WTAP and TNRC6 may be involved in those circRNA’s biogenesis.

### 3.6. The Expression of circNFIA and circZNF561 in Prostate Cancer

**Figure 6:**
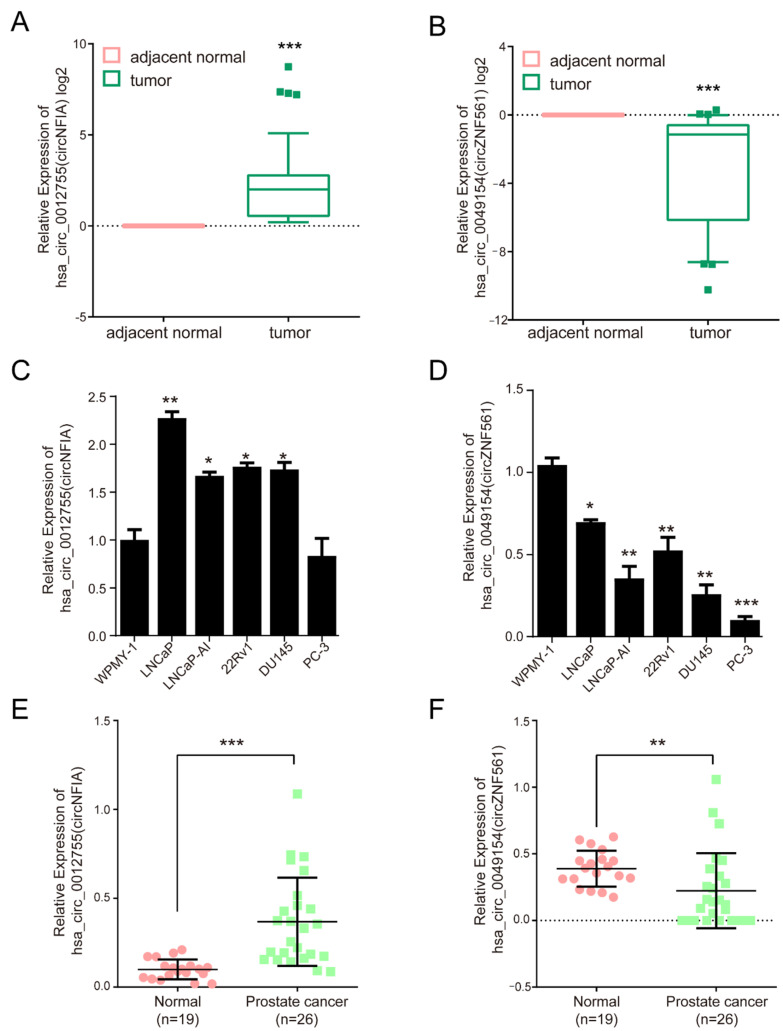
The expression of *circNFIA* and *circZNF561* in prostate cancer. (**A**,**B**) qPCR analysis of the expression of *circNFIA* and *circZNF561* in 30 pairs of prostate cancer tissue samples and corresponding adjacent tissue samples. (**C**,**D**) qPCR analysis of the expression of *circNFIA* and *circZNF561* in prostate cancer cell lines. (**E**,**F**) qPCR analysis of the expression of *circNFIA* and *circZNF561* in 26 prostate cancer plasma samples and 19 normal plasma samples. The *p*-value was calculated by the unpaired two-tailed Student’s *t*-test. * *p* < 0.05; ** *p* < 0.01; *** *p* < 0.001 (circRNAs).

To explore the biological roles of androgen-responsive circRNAs in prostate cancer, we identified differentially expressed circRNAs in 30 pairs of prostate cancer tissue samples and corresponding adjacent normal tissues by qPCR. Two circRNAs, *hsa-circ-0012755* (also called *circNFIA*) and *hsa-circ-0049154* (also called *circZNF561*), were significantly dysregulated in prostate cancer. The results showed that *circNFIA* expression was increased and *circZNF561* expression was decreased in prostate cancer tissue samples compared to adjacent normal tissues (Figure 6A,B). Furthermore, we also detected the expression of *circNFIA* and *circZNF561* in prostate cancer cells and plasma samples. As shown in Figure 6C,D, the expression of *circNFIA* was significantly upregulated in prostate cancer cell lines (LNCaP, LNCaP-AI, DU145, 22Rv1, and PC-3 cells), while *circZNF561* was downregulated in prostate cancer cells. The expression of *circNFIA* and *circZNF561* in plasma samples was further detected by qPCR. Consistent with the above results, *circNFIA* was significantly overexpressed in the plasma samples of prostate cancer patients, while the expression of *circZNF561* was decreased (Figure 6E,F). These results suggest that *circNFIA* and *circZNF561* may play roles in the progression of prostate cancer.

### 3.7. The Biological Functions of circNFIA and circZNF561 in Prostate Cancer

**Figure 7:**
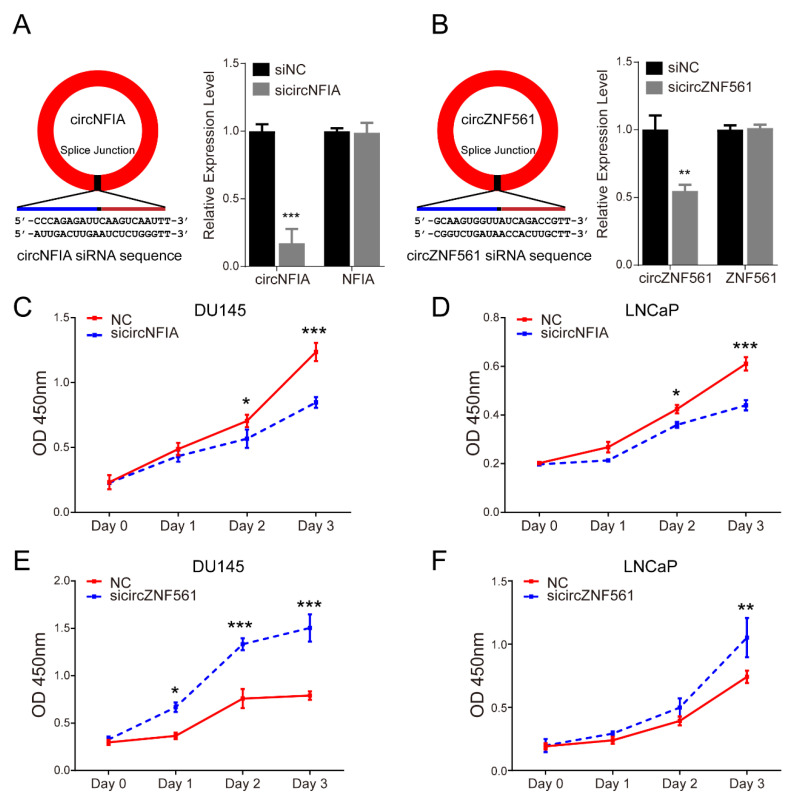
The biological function of *circNFIA* and *circZNF561***.** (**A**,**B**) siRNA was designed to specifically target *circNFIA* (A left) or *circZNF561* (B left). The efficiency of si*circNFIA* (A right) and *circZNF561* (B right) was confirmed by qPCR. (**C**–**F**) Cell proliferation analysis was performed with a CCK-8 assay in LNCaP and DU145 cells after knockdown of *circNFIA* and *circZNF561*. The *p*-value was calculated by the unpaired two-tailed Student’s *t*-test. * *p* < 0.05; ** *p* < 0.01; *** *p* < 0.001 (circRNAs).

Next, we knocked down *circNFIA* and *circZNF561* expression in the DU145 and LNCaP cell lines and explored their possible biological role in prostate cancer. As shown in Figure 7A,B, si*circNFIA* and si*circZNF561* significantly decreased the expression of *circNFIA* and *circZNF561*. The CCK-8 assay showed that knockdown of *circNFIA* significantly inhibited cell proliferation and si*circZNF561* promoted proliferation in prostate cancer (Figure 7C–F).

**Figure 8:**
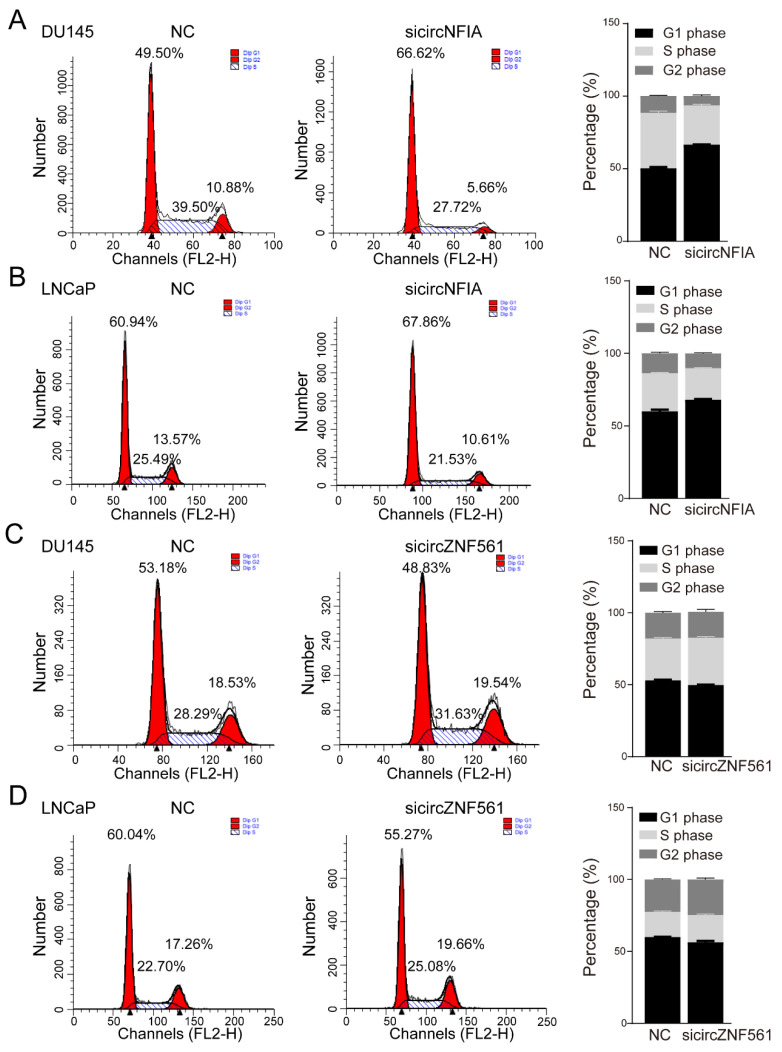
The biological function of *circNFIA* and *circZNF561*. (**A**–**D**) Cell cycle assay was performed in DU145 and LNCaP cells after knockdown of *circNFIA* and *circZNF561*.

In addition, we also tested the effect of knocking down *circNFIA* and circZNF561 on the cell cycle. The result showed that knocking down *circNFIA* increased the percentage of cells in the G1 phase (49.5 to 66.62% in DU145, 60.94 to 67.86% in LNCaP) and decreased the percentage of cells in the S phase (39.5 to 27.72% in DU145, 25.49 to 21.53% in LNCaP) to arrest cell cycle progression (Figure 8A,B). In contrast, si*circZNF561* decreased the percentage of cells in the G1 phase and increased the percentage of cells in the S phase, these results suggested si*circZNF561* could promote the cell cycle (Figure 8C,D).

**Figure 9:**
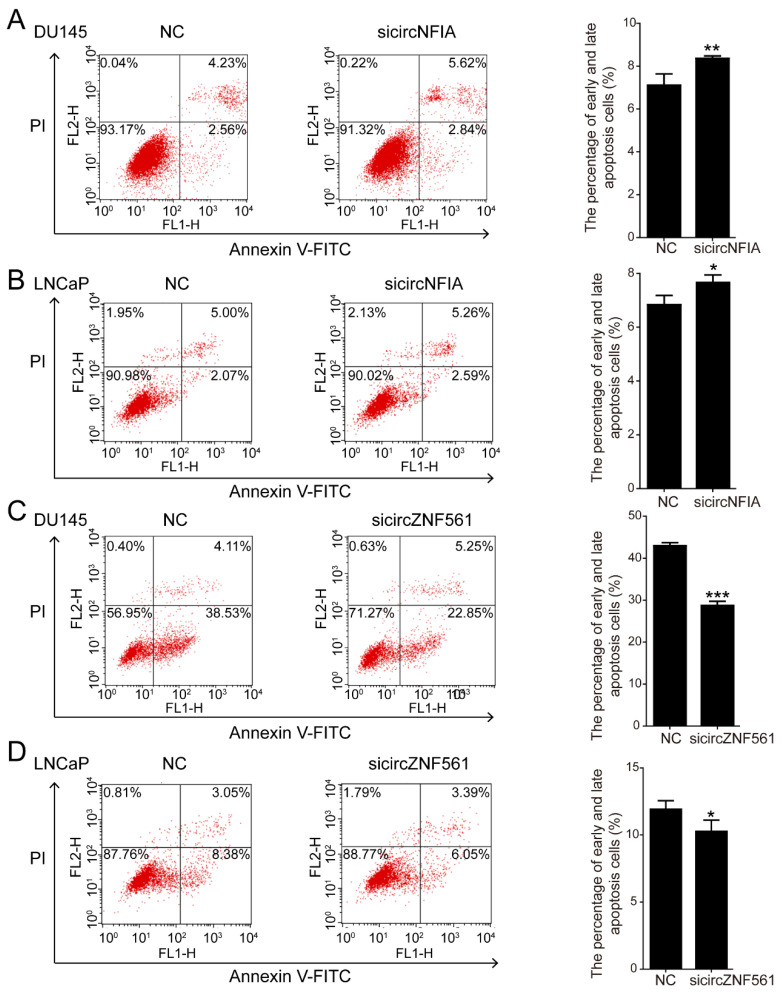
The biological function of *circNFIA* and *circZNF561***.** (**A**–**D**) Cell apoptosis assays were performed in DU145 and LNCaP cells after knockdown of *circNFIA* and *circZNF561*. The data are presented as the mean ± SD (*n* = 3). The *p*-value was calculated by the unpaired two-tailed Student’s *t*-test. * *p* < 0.05; ** *p* < 0.01; *** *p* < 0.001.

Similarly, apoptosis assays showed that si*circNFIA* significantly promoted cell apoptosis in DU145 and LNCaP cells. Compared with the control, apoptotic cells increased from 6.79% (DU145) and 7.07% (LNCaP) to 8.46% (DU145) and 7.85% (LNCaP) (Figure 9A,B). After knocking down circZNF561, we detected the percentage of apoptosis cells was decreased (Figure 9C,D). Taken together, we found that *circNFIA* may play an oncogenic role and *circZNF561* may act as a tumor suppressor in prostate cancer. These results indicated that *circNFIA* and *circZNF561* can be used as candidate tumor biomarkers.

### 3.8. Construction of a circRNA-miRNA ceRNA Network

**Figure 10:**
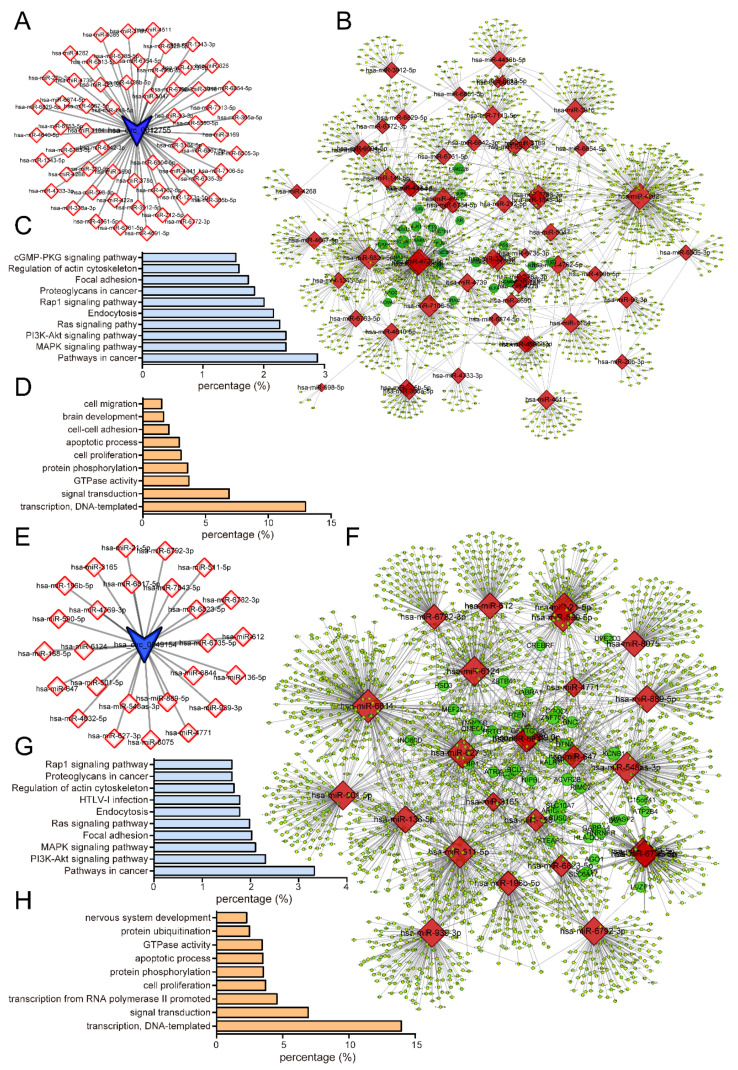
Construction of the *circNFIA*/*circZNF561*-mediated ceRNA network in prostate cancer. (**A**,**E**) Prediction of *circNFIA-* and *circZNF561*-targeted miRNAs by circBank. (**B**,**F**) Clusters of *circNFIA-* or *circZNF561*-mediated ceRNA networks in prostate cancer. The networks were drawn using Cytoscape 3.0. (**C**,**D**) GO and KEGG pathway analysis of target genes in *circNFIA*-mediated ceRNA networks. (**G**,**H**) GO and KEGG pathway analysis of target genes in *circZNF561*-mediated ceRNA networks.

As mentioned in previous reports, endogenous circRNAs could act as efficient microRNA sponges to regulate the expression of protein-coding genes [29,32]. To explore the mechanism by which *circNFIA* and *circZNF561* regulated prostate cancer progression, the putative candidate miRNAs binding to *circNFIA* or *circZNF561* were predicted using circBank. As shown in Figure 10A,E, we found 59 miRNAs targeted to *circNFIA* and 26 miRNAs targeted to *circZNF561*. Next, we constructed a circRNA-mediated competing endogenous RNA (ceRNA) network. Using the miRDB database, we identified the target genes of miRNAs interacting with *circNFIA* or *circZNF561*. The networks were drawn using Cytoscape 3.0 (Figure 10B,F)

Moreover, we performed Gene Ontology (GO) and KEGG pathway analysis of potential target genes in the network using the DAVID Bioinformatics Database (Figure 10C,D,G,H). Our results showed that target genes within the *circNFIA*-related network were mainly enriched in the cGMP-PKG, Rap1, Ras, PI3K-Akt and MAPK signaling pathways and were associated with regulating cell migration, cell-cell adhesion and apoptotic processes. (Figure 10C,D). The *circZNF561*-related network was mainly enriched in the Rap1, Ras, MAPK, and PI3K-Akt signaling pathways and was associated with regulating protein ubiquitination, GTPase activity, apoptotic processes, and cell proliferation (Figure 10G,H).

## 4. Discussion

ADT is the main treatment for prostate cancer, especially advanced and metastatic prostate cancer. With AR mutation or amplification, a low concentration of androgen can still activate the AR signaling pathway in CRPC, causing rapid worsening of prostate cancer in patients. Therefore, our group and others focused on exploring the important regulator of the AR signaling pathway in prostate cancer. Androgen-responsive noncoding RNAs, including miRNAs [33] and lncRNAs [31], have been reported, while androgen-responsive circRNAs have rarely been studied. In this study, we identified androgen-responsive circRNAs through high-throughput microarray analysis. The expression of six circRNAs (*hsa-circ-0076151*, *hsa-circ-0001055*, *hsa-circ-0005035*, *hsa-circ-0005079*, *hsa-circ-0008326*, and *hsa-circ-0073627*) regulated at the transcriptional level was validated by qPCR. Importantly, ChIP-seq data showed that the AR peaks in these circRNA loci significantly increased after DHT treatment, suggesting that these circRNAs were direct targets of AR.

Recent studies have reported that the biogenesis of circRNAs is mainly regulated by cis-elements or trans-factors, that is, through complementary base pairing on the flanking sequence [15,16] or binding to RBPs special motifs [34,35]. In this study, we identified RBPs involved in the biogenesis of circRNA using CircInteractome. We found that WTAP mainly participated in the formation of androgen-reduced circRNAs, while TNRC6 mainly participated in androgen-induced circRNAs. To further explore the importance of WTAP/TNRC6 in the biogenesis of circRNAs, we knocked down WTAP or TNRC6 and detected candidate circRNAs expression. The expression of *hsa_circ_0012079*, *hsa_circ_0087714*, and *hsa_circ_0018744* was significantly decreased after WTAP knockdown. Similarly, the expression of *hsa_circ_0090744*, *hsa_circ_0076815*, *hsa_circ_0049519*, *hsa_circ_0014100*, and *hsa_circ_0061590* was significantly decreased after TNRC6 knockdown. These results indicated that WTAP and TNRC6 might both involve in circRNA processing.

CircRNAs were more stable and tissue-specific than linear RNAs. Recent studies have reported that circRNAs could act as promising noninvasive markers for cancer diagnosis and prognosis [36,37]. In addition, abnormal expression of circRNAs has been observed in multiple types of cancer. In this study, we found that two circRNAs, *circNFIA* and *circZNF561*, were significantly differentially expressed in prostate cancer cells, tissue samples and plasma samples from patients with prostate cancer. Our results showed that *circNFIA* was significantly upregulated in prostate cancer cells, prostate cancer tissues, and plasma samples, while *circZNF561* was downregulated in prostate cancer cells, tissues and plasma samples. Therefore, *circNFIA* and *circZNF561* are potential diagnostic markers in prostate cancer.

Many studies have reported that circRNA expression is related to the etiology and progression of cancer and is involved in regulating the key signaling pathway of cancers [38]. To characterize the biological roles of *circNFIA* and *circZNF561*, we knocked down *circNFIA* and *circZNF561* and explored the effect on cell proliferation, cell cycle, and cell apoptosis. In this study, we revealed that *circNFIA* may play an oncogenic role, while *circZNF561* may act as a tumor suppressor in prostate cancer.

As mentioned in previous reports, circRNAs can act as miRNA sponges to regulate target genes and affect tumor progression. Wei et al. reported that *CDR1as* inhibited the tumor suppressor *miR-7* to promote cell proliferation in NSCLC [39]. Zhang et al. reported that *circFOXO3* can act as a *miR-138-5p* and *miR-432-5p* sponge to promote the expression of NFAT5 and promote the progression of glioblastoma [40]. *CircFOXO3* could also promote the occurrence and development of prostate cancer by competitively binding *miR-29a-3p* [29]. To explore the potential mechanism by which *circNFIA* and *circZNF561* played a role in prostate cancer, we constructed *circNFIA-* or *circZNF561*-mediated ceRNA networks by using the circBank and miRDB databases. We demonstrate that this study provides useful information for exploring potential therapeutic and prognostic biomarkers for prostate cancer.

## 5. Conclusions

In conclusion, we performed a androgen-responsive circRNAs analysis in prostate cancer patient tissues and identified two important regulatory factors, WTAP and TNRC6, which may involve in circRNA biogenesis. In addition, we explored the potential biological functions and predicted the molecular mechanism of two androgen-responsive circRNAs (*circNFIA* and *circZNF561*) by computational analysis and experimental verification, which may act as promising biomarkers for the diagnosis and treatment of prostate cancer.

## Data Availability

Not applicable.

## Supplementary Materials

The following are available online at https://www.mdpi.com/article/10.3390/life11101096/s1, Table S1: Sequence information of primers.

## References

[1] Sung H., Ferlay J., Siegel R.L., Laversanne M., Soerjomataram I., Jemal A., Bray F. (2021). Global Cancer Statistics 2020: GLOBOCAN Estimates of Incidence and Mortality Worldwide for 36 Cancers in 185 Countries. CA Cancer J. Clin..

[2] Center M.M., Jemal A., Lortet-Tieulent J., Ward E., Ferlay J., Brawley O., Bray F. (2012). International variation in prostate cancer incidence and mortality rates. Eur Urol..

[3] Huggins C., Hodges C.V. (1972). Studies on prostatic cancer. I. The effect of castration, of estrogen and androgen injection on serum phosphatases in metastatic carcinoma of the prostate. CA Cancer J. Clin..

[4] Scher H.I., Liebertz C., Kelly W.K., Mazumdar M., Brett C., Schwartz L., Kolvenbag G., Shapiro L., Schwartz M. (1997). Bicalutamide for advanced prostate cancer: The natural versus treated history of disease. J. Clin. Oncol..

[5] Chandrasekar T., Yang J.C., Gao A.C., Evans C.P. (2015). Mechanisms of resistance in castration-resistant prostate cancer (CRPC). Transl. Androl. Urol..

[6] Kelly W.K., Scher H.I. (1993). Prostate specific antigen decline after antiandrogen withdrawal: The flutamide withdrawal syndrome. J. Urol..

[7] Visakorpi T., Hyytinen E., Koivisto P., Tanner M., Keinanen R., Palmberg C., Palotie A., Tammela T., Isola J., Kallioniemi O.P. (1995). In vivo amplification of the androgen receptor gene and progression of human prostate cancer. Nat. Genet..

[8] Gregory C.W., Johnson R.J., Mohler J.L., French F.S., Wilson E.M. (2001). Androgen receptor stabilization in recurrent prostate cancer is associated with hypersensitivity to low androgen. Cancer Res..

[9] Makridakis N.M., di Salle E., Reichardt J.K. (2000). Biochemical and pharmacogenetic dissection of human steroid 5 alpha-reductase type II. Pharmacogenetics.

[10] Wang Q., Carroll J.S., Brown M. (2005). Spatial and temporal recruitment of androgen receptor and its coactivators involves chromosomal looping and polymerase tracking. Mol. Cell.

[11] Chung A.C., Zhou S., Liao L., Tien J.C., Greenberg N.M., Xu J. (2007). Genetic ablation of the amplified-in-breast cancer 1 inhibits spontaneous prostate cancer progression in mice. Cancer Res..

[12] Wang Q., Li W., Zhang Y., Yuan X., Xu K., Yu J., Chen Z., Beroukhim R., Wang H., Lupien M. (2009). Androgen receptor regulates a distinct transcription program in androgen-independent prostate cancer. Cell.

[13] Geller J., Albert J., Loza D. (1979). Steroid levels in cancer of the prostate—Markers of tumour differentiation and adequacy of anti-androgen therapy. J. Steroid. Biochem..

[14] Mostaghel E.A. (2014). Abiraterone in the treatment of metastatic castration-resistant prostate cancer. Cancer Manag. Res..

[15] Ivanov A., Memczak S., Wyler E., Torti F., Porath H.T., Orejuela M.R., Piechotta M., Levanon E.Y., Landthaler M., Dieterich C. (2015). Analysis of intron sequences reveals hallmarks of circular RNA biogenesis in animals. Cell Rep..

[16] Kelly S., Greenman C., Cook P.R., Papantonis A. (2015). Exon Skipping Is Correlated with Exon Circularization. J. Mol. Biol..

[17] Zhang X.O., Wang H.B., Zhang Y., Lu X., Chen L.L., Yang L. (2014). Complementary sequence-mediated exon circularization. Cell.

[18] Chen S., Li T., Zhao Q., Xiao B., Guo J. (2017). Using circular RNA hsa_circ_0000190 as a new biomarker in the diagnosis of gastric cancer. Clin. Chim. Acta..

[19] Xu H., Guo S., Li W., Yu P. (2015). The circular RNA Cdr1as, via miR-7 and its targets, regulates insulin transcription and secretion in islet cells. Sci. Rep..

[20] Wang K., Long B., Liu F., Wang J.X., Liu C.Y., Zhao B., Zhou L.Y., Sun T., Wang M., Yu T. (2016). A circular RNA protects the heart from pathological hypertrophy and heart failure by targeting miR-223. Eur. Heart J..

[21] Miao Q., Zhong Z., Jiang Z., Lin Y., Ni B., Yang W., Tang J. (2019). RNA-seq of circular RNAs identified circPTPN22 as a potential new activity indicator in systemic lupus erythematosus. Lupus.

[22] Lukiw W.J. (2013). Circular RNA (circRNA) in Alzheimer’s disease (AD). Front. Genet..

[23] Yu J., Xu Q.G., Wang Z.G., Yang Y., Zhang L., Ma J.Z., Sun S.H., Yang F., Zhou W.P. (2018). Circular RNA cSMARCA5 inhibits growth and metastasis in hepatocellular carcinoma. J. Hepatol..

[24] Barbagallo D., Caponnetto A., Cirnigliaro M., Brex D., Barbagallo C., D’Angeli F., Morrone A., Caltabiano R., Barbagallo G.M., Ragusa M. (2018). CircSMARCA5 Inhibits Migration of Glioblastoma Multiforme Cells by Regulating a Molecular Axis Involving Splicing Factors SRSF1/SRSF3/PTB. Int. J. Mol. Sci..

[25] Wang Y., Li H., Lu H., Qin Y. (2019). Circular RNA SMARCA5 inhibits the proliferation, migration, and invasion of non-small cell lung cancer by miR-19b-3p/HOXA9 axis. Onco. Targets Ther..

[26] Kong Z., Wan X., Zhang Y., Zhang P., Zhang Y., Zhang X., Qi X., Wu H., Huang J., Li Y. (2017). Androgen-responsive circular RNA circSMARCA5 is up-regulated and promotes cell proliferation in prostate cancer. Biochem. Biophys. Res. Commun..

[27] Lou J., Hao Y., Lin K., Lyu Y., Chen M., Wang H., Zou D., Jiang X., Wang R., Jin D. (2020). Circular RNA CDR1as disrupts the p53/MDM2 complex to inhibit Gliomagenesis. Mol. Cancer..

[28] Hanniford D., Ulloa-Morales A., Karz A., Berzoti-Coelho M.G., Moubarak R.S., Sanchez-Sendra B., Kloetgen A., Davalos V., Imig J., Wu P. (2020). Epigenetic Silencing of CDR1as Drives IGF2BP3-Mediated Melanoma Invasion and Metastasis. Cancer Cell.

[29] Kong Z., Wan X., Lu Y., Zhang Y., Huang Y., Xu Y., Liu Y., Zhao P., Xiang X., Li L. (2020). Circular RNA circFOXO3 promotes prostate cancer progression through sponging miR-29a-3p. J. Cell Mol. Med..

[30] Zhang C., Han X., Yang L., Fu J., Sun C., Huang S., Xiao W., Gao Y., Liang Q., Wang X. (2020). Circular RNA circPPM1F modulates M1 macrophage activation and pancreatic islet inflammation in type 1 diabetes mellitus. Theranostics.

[31] Wan X., Huang W., Yang S., Zhang Y., Pu H., Fu F., Huang Y., Wu H., Li T., Li Y. (2016). Identification of androgen-responsive lncRNAs as diagnostic and prognostic markers for prostate cancer. Oncotarget.

[32] Hansen T.B., Jensen T.I., Clausen B.H., Bramsen J.B., Finsen B., Damgaard C.K., Kjems J. (2013). Natural RNA circles function as efficient microRNA sponges. Nature.

[33] Mo W., Zhang J., Li X., Meng D., Gao Y., Yang S., Wan X., Zhou C., Guo F., Huang Y. (2013). Identification of novel AR-targeted microRNAs mediating androgen signalling through critical pathways to regulate cell viability in prostate cancer. PLoS ONE.

[34] Conn S.J., Pillman K.A., Toubia J., Conn V.M., Salmanidis M., Phillips C.A., Roslan S., Schreiber A.W., Gregory P.A., Goodall G.J. (2015). The RNA binding protein quaking regulates formation of circRNAs. Cell.

[35] Errichelli L., Dini M.S., Laneve P., Colantoni A., Legnini I., Capauto D., Rosa A., De Santis R., Scarfo R., Peruzzi G. (2017). FUS affects circular RNA expression in murine embryonic stem cell-derived motor neurons. Nat. Commun..

[36] Li Y., Zheng Q., Bao C., Li S., Guo W., Zhao J., Chen D., Gu J., He X., Huang S. (2015). Circular RNA is enriched and stable in exosomes: A promising biomarker for cancer diagnosis. Cell Res..

[37] Vo J.N., Cieslik M., Zhang Y., Shukla S., Xiao L., Zhang Y., Wu Y.M., Dhanasekaran S.M., Engelke C.G., Cao X. (2019). The Landscape of Circular RNA in Cancer. Cell.

[38] Kristensen L.S., Hansen T.B., Veno M.T., Kjems J. (2018). Circular RNAs in cancer: Opportunities and challenges in the field. Oncogene.

[39] Zhang X., Yang D., Wei Y. (2018). Overexpressed CDR1as functions as an oncogene to promote the tumor progression via miR-7 in non-small-cell lung cancer. Onco. Targets Ther..

[40] Zhang S., Liao K., Miao Z., Wang Q., Miao Y., Guo Z., Qiu Y., Chen B., Ren L., Wei Z. (2019). CircFOXO3 promotes glioblastoma progression by acting as a competing endogenous RNA for NFAT5. Neuro. Oncol..

